# Maternal Microbiome and Infections in Pregnancy

**DOI:** 10.3390/microorganisms8121996

**Published:** 2020-12-15

**Authors:** Mohammed Amir, Julia A. Brown, Stephanie L. Rager, Katherine Z. Sanidad, Aparna Ananthanarayanan, Melody Y. Zeng

**Affiliations:** 1 Gale and Ira Drukier Institute for Children’s Health, Weill Cornell Medicine, Cornell University, New York, NY 10021, USA; maa4012@med.cornell.edu (M.A.); jub4008@med.cornell.edu (J.A.B.); kzs4001@med.cornell.edu (K.Z.S.); apa4002@med.cornell.edu (A.A.); 2 Department of Pediatrics, Weill Cornell Medicine, New York, NY 10021, USA; slr4001@med.cornell.edu

**Keywords:** gut microbiome, pregnancy, infection, metabolism

## Abstract

Pregnancy induces unique changes in maternal immune responses and metabolism. Drastic physiologic adaptations, in an intricately coordinated fashion, allow the maternal body to support the healthy growth of the fetus. The gut microbiome plays a central role in the regulation of the immune system, metabolism, and resistance to infections. Studies have reported changes in the maternal microbiome in the gut, vagina, and oral cavity during pregnancy; it remains unclear whether/how these changes might be related to maternal immune responses, metabolism, and susceptibility to infections during pregnancy. Our understanding of the concerted adaption of these different aspects of the human physiology to promote a successful pregnant remains limited. Here, we provide a comprehensive documentation and discussion of changes in the maternal microbiome in the gut, oral cavity, and vagina during pregnancy, metabolic changes and complications in the mother and newborn that may be, in part, driven by maternal gut dysbiosis, and, lastly, common infections in pregnancy. This review aims to shed light on how dysregulation of the maternal microbiome may underlie obstetrical metabolic complications and infections.

## 1. Introduction

During pregnancy, the body undergoes major shifts in hormonal, metabolic, and immune regulation in order to promote healthy fetal development. In pregnant women, the necessity for the immune system to tolerate the growing fetus and unique tropism of the fetus for certain viruses result in an immunocompromised state that is vulnerable to infections. While the role of hormones during pregnancy has been well documented [1], how changes in the maternal microbiome are linked to various obstetrical conditions is less understood. A better understanding of how changes of the maternal microbiome may underlie the vulnerability of pregnant women to metabolic syndromes and infections will shed light on preventive measures that may be developed to improve the outcome of a pregnancy. Changes in the maternal microbiome occur beyond the gut, as there has been evidence linking oral bacteria and stillbirths and preterm births, vaginal dysbiosis, and preeclampsia. Obstetrical metabolic disorders, such as gestational diabetes, are common. Are they consequential to perturbed maternal microbiome? There has been mounting evidence to suggest against overuse of antibiotics in infants. Yet, the effects of prenatal antibiotic exposure on the newborn are still not well understood. In this review, we summarize recent research findings on these various topics related to the maternal microbiome. In addition, we survey common infections in pregnant women, their effects on the offspring, and current treatments.

## 2. Microbial Changes in the Maternal Microbiome during Pregnancy

### 2.1. Gut Microbiome

**Figure 1:**
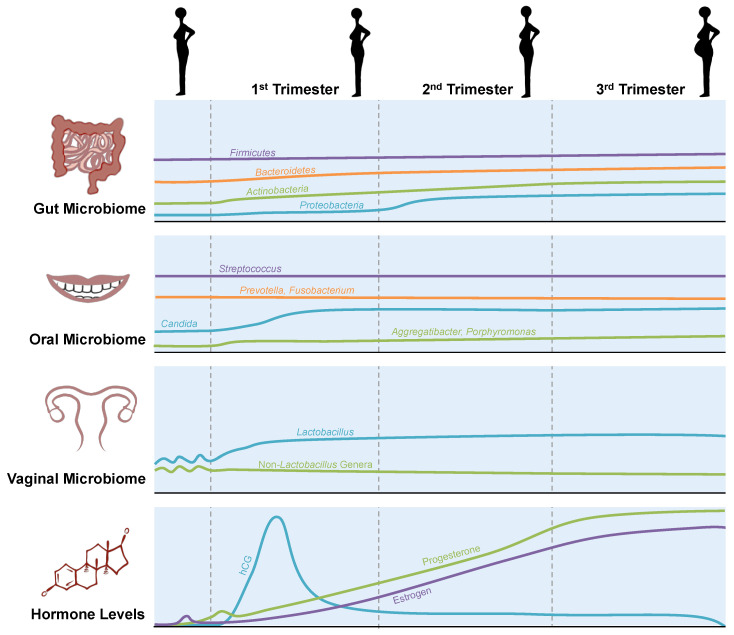
General changes in the gut, oral, and vaginal microbiomes in parallel with the hormonal changes throughout pregnancy. The changes that occur in the gut throughout pregnancy are complex and mediated by maternal factors, such as diet and antibiotic usage. The vaginal microbiome during pregnancy becomes more stably dominated by species of *Lactobacillus*, thereby decreasing in overall diversity.

Estrogen and progesterone have previously been shown to perturb the gut microbiome [2]. High levels of these hormones during pregnancy have been reported to increase the susceptibility of women to *Listeria monocytogenes* infection, which in turn may contribute to preterm delivery or stillbirth [3]. Progesterone can also directly influence the composition of the gut microbiome in pregnant women and lead to an increased abundance of *Bifidobacterium* [4]. Using culture-independent high-throughput sequencing, an increasing number of studies have reported changes in gut microbial diversity throughout the course of pregnancy and postpartum. From the first trimester to the third trimester, the relative abundance of *Proteobacteria* increased from 0.73% to 3.2% in almost 70% of pregnant women, while the abundance of *Actinobacteria* increased from 5.1% to 9.3% in 57% of pregnant women [5]. Interestingly, phylogenetic diversity decreased in the third trimester relative to the first trimester [5]. Members of *Clostridiales* appeared to bloom during the first trimester, whereas members of *Enterobacteriaceae* and *Streptococcus* expanded during the third trimester (Figure 1).

A mouse study of pregnancy showed that *Akkermansia* and *Bifidobacterium* species became most abundant during the early, but not late, gestational period [6]. *Bacteroides* species, however, increased in abundance early in gestation and remained elevated throughout the pregnancy. They also observed decreased abundance of *Coprobacillus*, *Clostridium,* and *Sarcina* during pregnancy, all of which belong to the *Firmicutes* phylum^6^. Some other studies, however, found no consistent changes in the gut microbiome during pregnancy or postpartum [7].

Additional evidence for microbial shifts during pregnancy comes from animal studies in which antibiotics were administered to pregnant animals. Mid-gestation administration of azithromycin, amoxicillin, and cefaclor led to an increased abundance of *Proteobacteria*, but significant reduction in *Lactobacillus* and *Firmicutes;* this reduction in bacterial diversity was associated with weight gain [8]. An interesting study reported gut bacteria from the third, rather than first, trimester, when transplanted into germ-free mice, induced metabolic changes resembling gestational diabetes [5]. This study underscored a major role for the gut microbiome in mediating metabolic changes during pregnancy. Microbial diversity during the postpartum period has not been studied extensively but could provide valuable information on the timeframe for microbial diversity to return to a nonpregnant state. One study indicates that, in humans, the mother’s microbiome remains altered for at least one month postpartum [5]. Hormonal and dietary changes during pregnancy underlie the changes in microbial richness and diversity.

### 2.2. Oral Microbiome

While the gut makes up the majority of the total microbiome, the oral cavity also acts as a diverse and rich reservoir of microbiota; housing over 700 species, the oral cavity has the second largest microbial diversity after the gut [9]. The resident microbial species in the oral cavity primarily belong to 12 phyla: *Actinobacteria*, *Bacteroidetes*, *Chlamydiae*, *Chloroflexi*, *Firmicutes*, *Fusobacteria*, *Gracilibacteria* (GN02), *Proteobacteria*, *Spirochaetes*, SR1, *Synergistetes*, and *Saccharibacteria* (TM7) [10]. A study of Japanese women observed an increased abundance of *Porphyromonas gingivalis* and *Aggregatibacter actinomycetemcomitans* during early and middle pregnancy, compared to non-pregnant groups, while *Candida* species were more abundant during middle and late pregnancy [11,12]. However, relatively little literature exists indicating how pregnancy-associated changes in the oral microbiome are induced, implying the need for more mechanistic studies in this area. Certain oral bacteria, such as *Campylobacter rectus*, *Fusobacterium nucleatum,* and *Porphyromonas gingivalis*, have been reported for their adverse effects on pregnancy [13,14,15] (Figure 1). Moreover, the presence of *F. nucleatum* in amniotic fluid cultures from patients who undergo preterm labor [13] suggests a possible transfer of oral bacteria to the placenta. However, that hypothesis raises the question of how oral bacteria traveling through the bloodstream are able to avoid detection by the immune system, and how these bacteria might affect the outcome of the pregnancy.

A case study of an unusual full-term stillbirth following maternal gingivitis found *F. nucleatum* present in both the placenta and the infant, which was found to have originated from the maternal subgingival plaque [16]. Fap2 of *F. nucleatum* was found to be a galactose-sensitive hemagglutinin and adhesin that contributes to the virulence of *F. nucleatum* for evasion of host immune surveillance and successful colonization in the placenta [17]. The case of *F. nucleatum* provides a mechanistic link between periodontal diseases and adverse pregnancy outcomes. Perhaps there are common environmental factors in the oral cavity and the placenta that promote the colonization and growth of certain bacteria, such as *F. nucleatum* [18]. Identifying the environmental uniqueness of the placenta to harbor these potentially pathogenic oral commensals will be an important step to understand how to protect pregnant women who develop periodontal diseases.

### 2.3. Vaginal Microbiome

Under normal physiological conditions, the vaginal microbiome is dominated by *Lactobacillus* species [19], including *Lactobacillus iners*, *L. crispatus, L. gasseri,* or *L. jensenii* [20], along with anaerobic bacterial communities, such as *Prevotella, Dialister, Atopobium, Gardnerella, Megasphaera, Peptoniphilus, Sneathia, Eggerthella, Aerococcus, Finegoldia*, and *Mobiluncus* [20,21,22,23,24,25]. The vaginal microbiome undergoes a shift in diversity during pregnancy [26], characterized by a decrease in diversity and richness in pregnant women, compared to non-pregnant subjects, with dominance of *Lactobacillus* species, *Clostridiales, Bacteroidales*, and *Actinomycetales*. Consistent with these findings, Romero and colleagues [27] also observed the dominance of *Lactobacillus* species during pregnancy, suggesting that the vaginal microbiome is relatively more stable during pregnancy than in the nonpregnant state.

Clinically, abnormal changes in the vaginal flora have been associated with preterm birth. *Gardnerella vaginalis,* for example, is associated with an increased risk of preterm labor, as is decreased frequency of *Lactobacillus* [28], while data from the Human Microbiome Project (HMP) indicated a lower abundance of vaginal *Lactobacillus crispatus* in cases of preterm birth [29]. However, in contrast, a study by Stout and colleagues failed to identify any unique taxa to be associated with preterm birth, but did observe decreased microbial diversity and richness in preterm subjects [30]. The cause of the low richness and diversity of the vaginal microbiome during pregnancy remains largely unclear, but some suggest it might be, in part, due to the rise in progesterone and estrogens [31]. The dominance of *Lactobacillus* species in the vaginal microbiome, and the resulting increase in acidity, might confer protection against urinary tract infections during pregnancy.

### 2.4. Effects of Maternal Dysbiosis during Pregnancy on the Offspring

Prolonged postnatal empirical antibiotic use is associated with changes in preterm gut microbiome and an increased risk of necrotizing enterocolitis (NEC), late onset sepsis (LOS), and death [32,33]. The effect of prenatal exposure on neonatal health is still not clearly understood, but maternal dysbiosis during pregnancy has been associated with a number of pregnancy complications, including preeclampsia, pre-term birth, and gestational diabetes [5,34,35,36,37,38,39,40,41,42,43]. Maternal dysbiosis may also have long-term effects on the offspring’s health; antibiotic use during pregnancy has been associated with the development of metabolic and allergic disorders later in childhood, including obesity and asthma infants [44,45,46].

In mice, antibiotic treatment during pregnancy can lead to defects in antiviral immune response in infants [47], as well as altered behavior and locomotive activity in the offspring at 4 weeks of age. Interestingly, behavioral defects can be rescued by fostering pups with untreated mothers. Moreover, a recently published study demonstrated that altering microbial composition during pregnancy through probiotic treatment prevented the development of obesity in both mothers and offspring [48], while another study observed that administering probiotics during pregnancy altered the expression of toll-like receptors (TLRs) in the placenta and in the meconium of the infant [49]. This could be of importance, given that appropriate microbial exposure during pregnancy can be beneficial in preventing allergies in offspring [50]. Recent studies have implicated a possible association between altered gut microbiome in children and increased risk of asthma and autism [51,52]. Collectively, these studies suggest that the maternal microbiome not only affects the success of a pregnancy, but may play an indispensable role in shaping the metabolism, behavior, and immunity of the offspring.

### 2.5. Metabolic Changes in Pregnancy and the Gut Microbiome

Gut bacteria have been known to play a role in energy extraction from food components, and thus directly impacting metabolism of the host [53]. Fiber-fermenting gut bacteria, such as *Bacteroides*, are crucial for metabolizing indigestible polysaccharides, regulating fat storage, and producing essential nutrients [53,54,55]. The metabolic shift that occurs during pregnancy is often seen as an adaptation to promote fetal growth and development. However, certain metabolic dysfunctions, such as insulin resistance, dyslipidemia, and hypertension, are common during the course of pregnancy [56]. Recent studies have suggested that changes to the microbiota during pregnancy may influence these pregnancy-associated metabolic changes. One particularly interesting study indicated that the transfer of third trimester maternal microbiome to non-pregnant germ-free mice resulted in the development of a metabolic syndrome characterized by high fat accumulation, weight gain, insulin resistance, and increased inflammatory response [5].

Other studies have established direct correlations between the abundance of *Collinsella* and circulating insulin, low-density lipoproteins, and triglycerides; between *Sutterella* and C-reactive protein; between *Lachnospiraceae* and leptin; and between *Bacteroidaceae* and ghrelin. Moreover, inverse correlations have also been reported between *Blautia* and insulin; between *Ruminococcaceae* and gastric inhibitory polypeptides (GIP); between *Prevotellaceae* and ghrelin level; between *Faecalibacterium:Fusobacterium* ratios and blood glucose levels; and between *Odoribacter* and arterial blood pressure [57], thus supporting the notion that pregnancy-induced microbial changes can potentially modulate the host metabolism by promoting enhanced absorption of glucose and fatty acids, and induction of catabolic pathways [5,58].

Not only do gut microbes modulate metabolism by directly facilitating energy absorption from food, but, interestingly, they can also regulate host metabolism through the secretion of extracellular vesicles. This is indicated by a study in which the transfer of extracellular vesicles derived from animals on a high-fat diet—in particular, extracellular vesicles derived from *Pseudomonas panacis* (phylum *Proteobacteria*)—induced insulin resistance and glucose intolerance in mice on a normal diet [59].

### 2.6. Changes in Gut Permeability during Pregnancy

The gut typically acts as a barrier preventing microbes and other intestinal contents from entering the bloodstream. However, mouse studies have found that during pregnancy, more molecules are able to cross this barrier. This loss of barrier function was demonstrated to be even greater when pregnant mice were fed a high-fat diet, resulting in increased inflammatory markers in maternal circulation. One study found that a maternal high-fat diet resulted in impaired gut barrier integrity, with corresponding increases in circulating maternal levels of pro-inflammatory bacterial lipopolysaccharides (LPS) and tumor necrosis factor (TNF) [60,61]. Placentas from dams fed a high-fat diet demonstrated blood vessel immaturity and hypoxia, decreased free carnitine, acylcarnitine derivatives, altered mRNA levels of inflammation, autophagy, and endoplasmic reticulum stress-associated genes. Fetuses born to dams fed a high-fat diet had increased activation of the pro-inflammatory transcription factor NF-κB, indicating a heightened state of immune activation [61]. Moreover, treatment of mice with prebiotic carbohydrates led to an increased abundance of *Bifidobacterium* species in the gut, resulting in reduced gut permeability [62].

## 3. Clinical Implications of the Gut Microbiome in Pregnancy

### 3.1. Gestational Diabetes

Pregnancy induces a metabolic shift to support the development of the fetus by promoting the uptake of glucose into adipose stores in preparation for the energy demands of later pregnancy, which leads to greater insulin resistance and, in some cases, gestational diabetes mellitus (GDM). GDM is characterized by any degree of new-onset glucose intolerance during pregnancy, and is one of the most common pregnancy complications [38]. Studies indicating that fecal transplants from third trimester mice could induce similar metabolic syndromes in non-pregnant mice [5] opened opportunities for further studies investigating the role of gut microbes in GDM. To this end, a cohort of individuals experiencing GDM in the third trimester showed enrichment of *Faecalibacterium* and *Anaerotruncus* species and depletion of *Clostridium* and *Veillonella*, compared to normoglycemic pregnant women in their third trimester [38]. Further studies found that women with GDM tended to have less microbial richness, compared to women without GDM, though the composition of their microbiomes did not differ significantly from that of matched controls [5]. Additionally, children born to mothers with GDM did not have significantly different microbiomes compared to children born to mothers without GDM, as measured by their Gini coefficients (a measure of “evenness”, or the disparity in relative abundances of species within a community) or overall microbial abundances.

Ultimately, more research is needed to assess any role the microbiome may play in the onset of GDM or any potential impacts of an altered microbiome on mothers and their children. Better understanding of these aspects of the condition may be important in preventing poor maternal and neonatal outcomes, such as cardiovascular disease, Type 2 diabetes, preeclampsia, and various neonatal respiratory and metabolic complications.

### 3.2. Preeclampsia

Preeclampsia is a life-threatening pregnancy complication known to affect 2–8% of pregnancies throughout the world [63], with nearly 300,000 cases annually in the United States [64]. It is a severe manifestation of placental dysfunction, likely due to early angiogenic and inflammatory dysregulation, and is characterized clinically by the new onset of hypertension, end organ dysfunction, and potential proteinuria after 20 weeks’ gestation. Pre-gestational diabetes, maternal obesity, multiple gestations, autoimmune disorders, and pre-existing high blood pressure or kidney disease are known to increase the risk of developing preeclampsia. However, because the precise cause of preeclampsia remains unclear, it is tempting to speculate that perturbations in the placental microbiome might also play a role in the development of the condition [65,66].

A 2014 study that analyzed placental samples from 110 women found that 12.7% of samples from women with preeclampsia contained bacteria, while all placental samples from matched normotensive primiparous women were PCR negative for the 16s rRNA gene, suggesting that the presence of bacteria in the placenta could be a risk factor for preeclampsia [67]. Interestingly, several of the bacterial species identified are typically associated with gut, respiratory tract, or periodontal infections. Another study by Barak et al. [68] identified the presence of *Actinobacillus actinomycestemcomitans, Fusobacterium nucleatum, Porphyromonas gingivalis, Prevotella intermedia, Tannerella forsynthia,* and *Treponema denticola* (Spirochaetes) in the placentas of women with preeclampsia. Strikingly, more than 50% of them were positive for periopathogenic organisms, indicating that periodontal disease may increase the risk of developing preeclampsia. Furthermore, maternal obesity is associated with gut dysbiosis and leaky gut syndrome, which could lead to enhanced inflammation, escape of bacteria from the gut, and resultant placental damage.

### 3.3. Fetal Growth Restriction

Fetal growth restriction (FGR), also known as intrauterine growth restriction (IUGR), is another relatively common and potentially devastating, yet poorly understood, obstetrical condition [69]. In FGR, the fetus is smaller than would be expected for its gestational age based on sonographic measurements. Clinically, it results from the inability of the fetus to maintain proper growth velocity, and is commonly defined as estimated weight below the 10^th^ percentile [70]. FGR may be a result of various maternal factors, such as age, infection, behavioral habits, genetic abnormalities, or insufficient delivery of nutrients by the placenta [71]. However, recent studies have also investigated whether changes in the microbiome may play a role in the pathogenesis of FGR.

A 2016 study of over 6000 pregnant women found that *Helicobacter pylori* colonization, particularly with inflammatory CagA strains, was positively associated with FGR [72]. Abnormal vaginal flora has also been associated with the development of FGR. A 2015 meta-analysis presents several studies that associated vaginal colonization by *U. urealyticum or M. hominis,* as well as bacterial vaginosis, with low birthweight [73]. The authors postulate that ascending genital infections may result in intrauterine inflammation and damage to trophoblasts, causing placental dysfunction and impaired fetal growth. Another study that examined the gut microbiome of piglets found that newborn piglets with IUGR had significantly lower bacterial diversity in the jejunum at 7 days of age [74]. Additionally, this study found that five bacterial taxa, including *Proteobacteria*, *Escherichia-Shigella*, *Pasteurella*, *Leptotrichia,* and *Erysipelothrix*, were negatively correlated with birth weight, and that there was a lower abundance of microbial pathways related to the metabolism of various types of macromolecules in piglets with IUGR. However, additional prospective cohort studies in humans are needed to confirm whether altered microbiomes are indeed a causative factor in the onset of FGR.

### 3.4. Infections during Pregnancy, Clinical Outcomes, and Therapeutics

It is relatively common for women to present with bacterial, viral, or parasitic infections during pregnancy. In fact, some studies suggest an increased susceptibility to certain infections during pregnancy as a result of compensatory immunologic changes [75]. Maternal infections require special attention (Table 1), as they present a risk of vertical transmission to the fetus, thus leading to adverse outcomes such as preterm birth, intrauterine growth restriction, developmental delays, and fetal demise. Here, we will discuss common infections in pregnant women, their effects on the offspring, currently available treatments, and several treatments currently under development.

### 3.5. Bacterial Infections

Bacterial infections during pregnancy can be caused by a single species of bacteria, by an imbalance in the microbiomes, or by gut dissemination of bacteria. Types of infections caused by specific bacteria include Group B Streptococcus (GBS) infection, listeriosis, urinary tract infections (UTIs), and some sexually transmitted infections (STIs), such as chlamydia, gonorrhea, and syphilis (Table 1). Bacterial vaginosis and uterine infections may also occur as a result of an imbalance of bacteria or by dissemination of bacteria into inappropriate regions.

GBS infections are caused by the bacteria *Streptococcus agalactiae,* which commonly lives in the gastrointestinal and genital tracts and can cause UTIs or bladder infections [122,123]. Compared to non-infected women, pregnant women infected with GBS have a greater risk of preterm labor [77], stillbirth [81], and vertical transmission to their babies [124]. GBS-infected infants can also develop a range of complications, including bacteremia [78], early onset or late-onset sepsis (EOS/LOS) [78], and meningitis [76]. GBS may also result in the death of infected neonates [125]. For these reasons, pregnant women are routinely tested for GBS during their third trimester so that IV penicillin G or ampicillin can be administered directly prior to labor and delivery [126].

Listeriosis is another infection that is more likely to occur in pregnant women and newborns than in non-pregnant women [127]. This infection is caused by the Gram-positive bacterium *Listeria monocytogenes*, which can be contracted after the consumption of contaminated food. Similarly to GBS, listeriosis can lead to preterm birth, stillbirth, miscarriages, meningitis, sepsis, and newborn death [83,127]. Fetal outcomes are especially bleak when the infection is blood-borne, with reports of greater than 50% fetal loss in this case [83]. Fortunately, listeriosis can be prevented simply by avoiding foods such as unpasteurized dairy, prepared deli meats and salads, hot dogs, and raw vegetables during pregnancy [83].

The STIs chlamydia (caused mainly by *Chlamydia trachomatis*) and gonorrhea (caused by *Neisseria gonorrhoeae*) have similar effects on infants who contract the infection from their mothers. These infections may result in preterm birth [92,95], low birth weight [128], and conjunctivitis that may lead to blindness [94]. Because it is often relatively asymptomatic, untreated chlamydia can progress to chronic pelvic inflammatory disease, which may cause fibrosis leading to pregnancy complications such as ectopic pregnancy with future conception [129,130]. Syphilis, another STI, is caused by the bacterium *Treponema pallidum*, and can lead to more complications in infants, including low birth weight, preterm birth, stillbirth, and newborn death [97].

Bacterial vaginosis (BV) is an infection resulting from dysbiosis in the vaginal microbiome [131] and is usually associated with a decrease of *Lactobacillus* species and an increase in anaerobes, such as *Gardnerella vaginalis* and *Mycoplasma hominis* [132]. Women with bacterial vaginosis have an increased risk of preterm labor and delivery of babies that are small for gestational age (SGA) [91]. BV during pregnancy may also result in chorioamnionitis, or intra-amniotic infection, which may endanger the fetus, due to increased inflammation in fetal membranes.

UTIs are another common infection in both pregnant and non-pregnant women. In both cases, the infection is usually the result of *Escherichia coli* and other Gram-negative rod bacteria [133]. While relatively benign in non-pregnant women, one study found that over 3% of antepartum hospital admissions were due to UTIs, as they can lead to adverse outcomes both in mothers (risk of preeclampsia, chorioamnionitis, and anemia) and infants (low birth weight and preterm birth) [133]. UTIs may also progress to intrauterine infections, potentially resulting in premature, dangerous, or difficult labor [134].

### 3.6. Viral Infections

The human microbiome contains a substantial viral component, including bacterial and archaeal viruses as well as viruses capable of infecting eukaryotic cells. The true extent of the human virome remains unknown, but some estimates place the viral component of the microbiome as outnumbering the bacterial/archaeal component by as much as 10 to 1 [135]. While the vast majority of viruses present in the human body are likely not harmful (and, in the case of archaeal viruses and bacteriophages, may help to keep the bacterial microbiome in check), there do exist a number of pathogenic viruses that have the potential to affect fetal or neonatal health. These include, among others, cytomegalovirus (CMV), hepatitis B (HBV), hepatitis C (HCV), rubella (RV), Zika virus (ZIKV), and viral STIs, such as the herpes simplex viruses (HSV-1 and HSV-2) and human immunodeficiency virus (HIV).

Cytomegalovirus (CMV) is a large, enveloped DNA virus that is a member of the beta herpesvirus subfamily of herpesviruses. It is transmitted from person to person via contact with infected bodily fluids, particularly saliva and urine, and can be passed from the mother to the fetus in utero [103]. CMV establishes lifelong persistent infection by entering a latent state within hematopoietic stem cells in the bone marrow [136]. However, it is the primary infection, not latent or reactivated virus, that poses the most danger during pregnancy; intrauterine transmission of CMV occurs in 40% of pregnancies in which a primary CMV infection occurs, compared to only 2% of pregnancies in which the mother experiences a reactivation of latent CMV [137]. One out of 200 babies are born with CMV, with potentially devastating consequences. CMV is the leading cause of infectious congenital malformations. One out of five CMV-infected babies will have long-term health problems, such as hearing loss (the most common sequela), vision loss, seizures, microcephaly, organ dysfunction, and intellectual disabilities [99,100]. Dermatologically, babies born with congenital CMV may also present with characteristic hemorrhagic purpura, colloquially referred to as ‘blueberry muffin syndrome’. This same patterning can also be seen in babies born with congenital rubella, herpes, toxoplasmosis, and other non-infectious conditions.

Similar to CMV, hepatitis B virus (HBV) is spread by contact with infected bodily fluids. Importantly, it has been found in amniotic fluid, vaginal fluid, and breastmilk, and thus there is a high risk for mother-to-infant transmission during delivery or in the first weeks of life. In fact, up to 30% of HBV cases result from mother-to-infant transmission [138]. Hepatitis C virus (HCV) is less readily transmitted from mother to infant, but vertical transmission of HCV is estimated to occur in 4–10% of infected mothers [139]. Both viruses can cause chronic liver disease or liver cancer in infected infants [105]. Fortunately, vaccination of neonates is highly effective in preventing mother-to-infant transmission of HBV, but no vaccine yet exists for HCV [138,140].

Rubella virus (RV) is part of the *Rubivirus* genus in the Togaviridae family of viruses, and causes a mild infection known as German measles (not to be confused with measles, which is caused by the measles virus (MV) in the paramyxovirus family) [107]. RV is primarily transmitted by respiratory droplets, but can cross the placental barrier by inducing necrosis of the syncytiotrophoblast layer [107,141]; transplacental transmission occurs in 50% of pregnancies complicated by RV infection, and this risk rises to nearly 90% when maternal infection occurs in the first trimester [106,107]. Congenital RV infection is associated with a wide range of ocular disorders, auditory problems, cardiovascular defects (in particular, patent ductus arteriosis), microcephaly, meningitis, encephalitis, speech disorders, motor defects, and autism [107,142].

Transmission of HIV from mother to child can occur throughout gestation, but the greatest risks are during delivery and breastfeeding [116]. In the absence of any intervention, the risk of vertical transmission of HIV during labor approaches 25%, and transmission via breastmilk occurs at similar rates [116,143]. Transmission of HIV to the infant places them at risk for developing acquired immunodeficiency syndrome (AIDS), necessitates lifelong antiretroviral treatment, and predisposes them to a number of significant health issues, including cardiovascular and liver disease [116].

HSV causes only mild symptoms in adults, with the majority of infections being asymptomatic, and latent HSV poses little risk to the infant [112]. However, a primary infection acquired close to the time of delivery carries a significant risk of transmission during labor, which can lead to herpes simplex encephalitis, a rare but severe disease with a mortality rate of 50–80% in the absence of treatment, and 4–30% with treatment [112].

Zika virus (ZIKV) has recently gained attention due to its ability to cross the placenta and cause devastating effects to the fetus. Zika virus is primarily transmitted by mosquitoes and causes very mild symptoms in adults, but infection during pregnancy increases the risk of miscarriage or stillbirth, and surviving infants can display significant neurological defects, including microcephaly, lissencephaly, brain calcifications, enlarged ventricles, collapsing brains, and asymmetrical brains [108,109,110]. The risk of birth defects is highest when infection occurs during the first trimester, and does not differ between symptomatic and asymptomatic maternal infections [144,145]. Several reports have found ZIKV RNA in placental tissue, amniotic fluid, and fetal brains [146,147].

### 3.7. Parasitic Infections

Other infections that may occur during pregnancy include toxoplasmosis and trichomoniasis, which are caused by the parasites *Toxoplasma gondii* (*T. gondii*) and *Trichomonas vaginalis*, respectively. *T. gondii* can be acquired through uncooked meat or contact with feces of infected felines [120]. Toxoplasma infections in pregnant women can result in miscarriages and stillbirth [119]. *T. gondii* can also cross the placenta and infect the fetus, causing intellectual disabilities, blindness, or other central nervous system (CNS) problems, though most infants are asymptomatic at birth [119]. Trichomoniasis is another STI that has been linked to increased risk of preterm birth in infected pregnant women [148].

### 3.8. Treatments and Therapeutics

There has been significant progress in the development of therapeutics to treat infections during pregnancy. Current guidelines recommend screening women for HIV, hepatitis, rubella, and common STIs early in pregnancy, and screening for GBS between weeks 36 and 38 of pregnancy [149,150]. With early intervention, some therapeutics, including vaccines, antibiotics, and antiviral drugs, have been proven effective. However, several infections do not yet have effective FDA-approved treatments, spurring current research efforts to develop novel therapies for these infections.

Studies have found that antibiotics are prescribed in around 25% of pregnancies. However, care must be taken to ensure that the type of antibiotic prescribed, and its timing during the course of the pregnancy, will not cause harm to the developing fetus [151]. Penicillin, cephalosporin, vancomycin, aztreonam, metronidazole, azithromycin, erythromycin, clindamycin, and daptomycin are generally considered safe for use during pregnancy [152]. Antibiotics that have been shown to have potentially adverse effects on offspring include streptomycin (implicated in congenital deafness if administered in the first trimester), tetracycline (linked to teratogenicity and maternal liver toxicity), tigecycline (resulted in fetal loss in animal studies), and folate antagonists (shown to increase the risk of neural tube and cardiac defects) [153].

For treatment of chlamydia and gonorrhea, newborns are given an antibiotic ointment, usually erythromycin, that is placed onto their eyes to prevent conjunctivitis [93]. Pregnant women with BV have been treated with metronidazole, clindamycin, and erythromycin, all of which have been shown to successfully eradicate BV [89]. However, only treatment with metronidazole and erythromycin decreased rates of premature deliveries [134]. Penicillin, one of the antibiotics most commonly prescribed during pregnancy, is given to treat syphilis, and antibiotic cocktails are given for treatment of GBS and listeriosis to help prevent infection and sepsis in infants [78,84,154]. For UTIs, the choice of antibiotics given are dependent on the bacteria that has caused the infection. Because 80–90% of UTIs are *E. coli*, ampicillin is usually given [133]. However, alternative antibiotics are continually being sought out, due to increased bacterial resistance to commonly prescribed drugs.

Vaccines and antiviral drugs are effective in treating and preventing many viral infections. For treatment of HBV infections, infants will receive the HBV vaccine along with hepatitis B immunoglobulin as soon as 12 h after birth [104]. The measles, mumps, and rubella (MMR) vaccine is highly effective against those diseases, and is commonly given to young infants [155]. There are currently various antiviral drugs that can be administered for the treatment of CMV, including ganciclovir, valganciclovir, foscarnet, and cidofovir, though these drugs are not necessarily recommended for use during pregnancy, due to limited safety and efficacy data [156]. Prophylactic antiretroviral therapy, with drugs such as zidovudine, is effective at reducing the rate of vertical transmission of HIV when given throughout pregnancy and the breastfeeding period; delivery via Caesarean section can also reduce the risk of transmission [115,118]. Similarly, antiviral drugs, such as acyclovir and valacyclovir, may be given to mothers with HSV, and Caesarean delivery is implicated to reduce chances of HSV infection in infants [111,157].

There are several treatments that are currently in development to decrease infection rates in both mothers and infants during the perinatal period. Although GBS infections can be treated with antibiotics, the rise in antibiotic resistance and the severity of neonatal GBS infections has led to efforts to develop a vaccine for GBS [158,159]. Vaccine development is also currently underway to prevent congenital CMV, given the severity of the effects of congenital CMV on the offspring [160,161]. There have also been interesting new developments in vaccines for Zika. Recently, a mouse model of Zika virus infection during pregnancy was developed to help aid in finding possible vaccines to protect against infection [162]. Additionally, there have been several studies that proposed possible Zika vaccine candidates (an attenuated virus vaccine, a DNA vaccine, and an inactivated virus vaccine) that can induce immunity in different mouse models of Zika infection [163,164].

## 4. Limitations in Animal Models of Pregnancy

It should be noted that many of the studies described in this article make use of rodent models of pregnancy. While small rodents are useful for obstetrical research because of their short gestational periods, relatively large litter sizes, and ease of manipulation, there are limitations with these rodent models, due to inherent differences between rodent and human pregnancies [165]. For example, rodents are born developmentally less mature than humans [166]. There are also differences in hormone production throughout mouse and human pregnancies [167]. In humans, progesterone is first produced by the corpus luteum, then the placenta, and levels of the hormone rise until delivery. However, in mice, progesterone synthesis decreases, and progesterone metabolism increases, in the last two days of pregnancy. In addition, in mice, estrogen levels are low in the first half of pregnancy, then increase dramatically in the second half of pregnancy, while estrogen increases more gradually throughout human pregnancies. These hormonal differences may have effects on the composition of the microbiome and susceptibility to various obstetrical conditions. There are also key differences in the structure of the human and murine placentas: the human placenta is monochorial, meaning that there is a single layer of cells (the syncytiotrophoblasts) separating maternal blood from fetal blood, while the mouse placenta is trichorial, with a layer of mononuclear trophoblasts lining maternal blood spaces followed by two layers of syncytiotrophoblasts that surround the fetal endothelium [168]. The mouse embryo is also surrounded by an extra placental layer, the choriovitelline placenta (or yolk sac), which—together with the extra layers of trophoblasts—has important implications regarding the interactions between maternal immune cells and the developing fetus, as well as the ability of pathogens to cross the placenta [168,169]. Some obstetric conditions, such as preeclampsia, cannot be replicated in animals using chemicals or genetic manipulations [170]. However, there remain many important similarities between rodent and human pregnancies, such as similar cardiovascular adaptations, and the relative roles of uterine and blood natural killer (NK) cells in promoting fetal survival or fetal loss [171]. Animal models of pregnancy remain valuable in research efforts to experimentally refine and test hypotheses that cannot be performed practically in humans.

## 5. Conclusions

Almost every organ system undergoes significant physiological changes during pregnancy. The maternal microbiome is no exception. Changes in both the diversity and composition of the oral, vaginal, and gut microbiomes during pregnancy may have a profound effect on maternal metabolism and immune responses; this, however, remains poorly understood. Common obstetrical conditions might, in part, attribute to dysregulation of maternal microbiome and subsequent impact on metabolism and immune responses. In addition, hormonal and immune changes in pregnant women, and unique tropism of the fetus for certain viruses, render pregnant women more susceptible to pathogenic or opportunistic infections. It is important to screen pregnant women for STIs, UTIs, food-borne illnesses, and Group B Streptococcus, which may increase risk for preterm birth or congenital malformations. A better understanding of dysregulation of maternal microbiome to susceptibility to these infectious pathogens may shed light on diagnostic or preventive measures to improve both maternal and neonatal health. 

## Figures and Tables

**Table 1:** List of common infections in pregnancy and associated effects.

Infection	Causative Agent(s)	Risk Factors	Effects on Offspring	Treatment	References
Group B Strep	*Streptococcus* *agalactiae*	History of GBS colonization in a previous pregnancy, nulliparity	Preterm birth, stillbirth, bacteremia, EOS or LOS, meningitis, death	Oral antibiotics (penicillin G), IV antibiotics during labor Newborns: supportive therapy, antibiotics	[76,77,78,79,80,81,82]
Listeriosis	*Listeria* *monocytogenes*	Ingestion of high-risk foods during pregnancy, including unpasteurized dairy, prepared deli meats and salads, hot dogs, and raw vegetables	Preterm birth, stillbirth, miscarriages, meningitis, sepsis, and newborn death	Penicillin, ampicillin, or amoxicillin	[83,84,85]
UTI	*Escherichia coli, Klebsiella pneumoniae,* other gram negative rods	Sexual activity, history of UTIs, bacterial vaginosis	If untreated: increased risk of preterm birth, low birth weight, and perinatal death	Nitrofurantoin, sulfisoxazole (not for use near term), cephalexin, or fosfomycin	[86,87,88]
Bacterial Vaginosis	Increased *Gardnerella vaginalis* and *Mycoplasma hominis,* decreased *Lactobacillus*	Multiple sex partners, douching, use of scented soap	Preterm labor, low birth weight, risk of miscarriage from chorioamnionitis	Metronidazole, clindamycin, or erythromycin	[89,90,91]
Chlamydia & Gonorrhea	Chlamydia: *Chlamydia trachomatis* *Gonorrhea: Neisseria gonorrhoeae*	Multiple sex partners, previous STI, inconsistent use of barrier protection	Preterm birth, low birth weight, conjunctivitis that may lead to blindness	Newborns: topical ocular prophylaxis with erythromycin	[92,93,94,95]
Syphilis	*Treponema pallidum*	Multiple sex partners, previous STI	Preterm birth, stillbirth, low birth weight	Penicillin G	[96,97,98]
CMV	Human cytomegalovirus	Primary infection during pregnancy	Hearing and/or vision loss, intellectual disabilities, microcephaly, hepatosplenomegaly, seizures, perinatal death	Supportive therapy: antiviral therapies, such as ganciclovir or valaciclovir, may be contraindicated during pregnancy but may be used to treat newborns	[99,100,101,102,103]
HBV	Hepatitis B virus	Contact with infected bodily fluids	Chronic liver disease or liver cancer	Newborns: HBV vaccine and Hep B immunoglobulin	[104,105]
RV	Rubella virus	Close contact with an infected individual	Ocular defects, auditory problems, cardiovascular defects, microcephaly, meningitis, encephalitis, neurodevelopmental delays; highest risk during first trimester infection	Supportive therapy; pregnancy counseling	[106,107]
ZIKV	Zika virus	Mosquito bites in endemic area	Miscarriage, stillbirth, microcephaly, lissencephaly, brain calcifications, enlarged ventricles, collapsing brain, and asymmetrical brain	Supportive therapy; close monitoring for fetal abnormalities	[108,109,110]
HSV	Herpes simplex virus	Genital herpes, early age of coitarche, more than one lifetime partner, previous genital chlamydia	Little effect from latent infection; risk of herpes simplex encephalitis, seizures, perinatal death from primary infection acquired close to delivery	Caesarian delivery, IV acyclovir prophylaxis Newborns: acyclovir if third-trimester primary infection and Caesarian delivery not performed	[111,112,113,114]
HIV	Human immunodeficiency virus	Inconsistent use of barrier protection, multiple sex partners, IV drug use, limited prenatal care	Risk of developing AIDS, cardiovascular and liver disease	Cesarean delivery, bottle feeding Newborns: Prophylactic antiretroviral therapy (zidovudine)	[115,116,117,118]
Toxoplasmosis	*Toxoplasma gondii*	Ingestion of undercooked meat, contact with feces of felines	Increased risk of severe disease if acquired during first trimester; Miscarriage, stillbirth, visual impairment, intellectual disability	Spiramycin for fetal prophylaxis; pyrimethamine, sulfadiazine, or folic acid for confirmed fetal infection	[119,120,121]
